# A positive association between phosphorylated Tau217 (pTau217) and neural correlations is prevented by human leukocyte antigen allele DRB1*13:01

**DOI:** 10.1038/s41598-026-44894-7

**Published:** 2026-03-26

**Authors:** Lisa M. James, George Stratigopoulos, Arthur C. Leuthold, Apostolos P. Georgopoulos

**Affiliations:** 1 The Healthy Brain Aging Group and the Domenici Research Center for Mental Illness, Brain Sciences Center, Department of Veterans Affairs Health Care System, Minneapolis (Minneapolis VAHCS), One Veterans Drive, Minneapolis, MN 55417 USA; 2 Department of Neuroscience, University of Minnesota Medical School, Minneapolis, MN USA; 3 Department of Psychiatry, University of Minnesota Medical School, Minneapolis, MN USA; 4 Institute for Health Informatics, University of Minnesota Medical School, Minneapolis, MN USA

**Keywords:** Dementia blood biomarkers, pTau217, Viruses, Human leukocyte antigen (HLA), Apolipoprotein E, Magnetoencephalography, Synchronous neural interactions, Biomarkers, Diseases, Genetics, Immunology, Microbiology, Neurology, Neuroscience

## Abstract

Subtle deviations in zero-lag cross correlations (synchronous neural interactions, SNI) differentiate healthy from pathological brain states. Here, we assessed blood biomarkers of dementia and neurodegeneration in relation to SNI in 175 women participating in a longitudinal study for a total of 348 acquisitions. Of seven biomarkers tested (Aβ_1–40_, Aβ_1–42_, Aβ_1–42_/Aβ_1–40_ ratio, neurofilament light chain [NFL], total Tau, phosphorylated Tau181 [pTau181], phosphorylated Tau217 [pTau217]), only pTau217 showed a significant positive association with SNI (*P* = 0.0027) which was negatively associated with cognitive performance. Apolipoprotein E (ApoE) did not influence the pTau217-SNI association, whereas human leukocyte antigen (HLA) DRB1*13:01 reduced the association. The pTau217-SNI association was pronounced in participants seropositive for human herpes virus 1 (HHV1) and human endogenous retrovirus K (HERVK). This effect was abolished in carriers of HLA DRB1*13:01, consistent with the strong predicted binding affinity of this allele with proteins of these viruses. These findings highlight the association of pTau217 with brain function and point to a central role of HLA-mediated immune responses in preserving brain health.

**Supplementary Information:**

The online version contains supplementary material available at 10.1038/s41598-026-44894-7.

## Introduction

### Magnetoencephalography (MEG) and cognitive function

Dynamic communication across the massive, interconnected network of neurons lies at the heart of brain function. Magnetoencephalography (MEG) measures the magnetic fields generated by motion of ions across cell membranes associated with excitatory synapses along the dendrites of pyramidal cells in the cerebral cortex, providing a direct, non-invasive measure of neuronal activity with millisecond temporal resolution^1,2^. MEG has been instrumental in distinguishing healthy brain function from anomalies associated with conditions affecting the brain including cognitive decline^3–11^. Previous research has shown that subtle decrements in cognitive performance are significantly associated with increased synchronous neural interactions (SNI), even among cognitively healthy individuals, whereas high performance on cognitive screening measures is associated with neural network decorrelation^11^. Given the relevance of neural decorrelation for information processing^12,13^, subtle increases in SNI may portend cognitive impairment associated with restriction in dynamic communication across neural ensembles that impact information processing^11^, the progression of which may be associated with objective cognitive decline and neurodegeneration.

### Blood biomarkers for dementia

Blood biomarkers have emerged as a promising tool for detecting proteins that are associated with neurodegeneration and dementia^14,15^. With the advent of ultrasensitive techniques, biomarkers obtained from the blood have proven to be highly accurate, approaching the accuracy of cerebrospinal fluid (CSF) biomarkers^16^. The most commonly studied biomarkers include those evaluating amyloid-beta (Aβ_1–40_ [Aβ40], Aβ_1–42_ [Aβ42], Aβ42/Aβ40 ratio) and phosphorylated tau (pTau181, pTau217) proteins, which are commonly associated with Alzheimer’s dementia, as well as markers of neurodegeneration including total tau and neurofilament light chain (NFL)^17–20^. Remarkably, an increase in biomarker levels can precede dementia diagnosis by up to 20 years^21,22^, highlighting dementia-related brain changes that occur well in advance of overt disease. Here, in a sample of non-demented women, we evaluated the association of blood biomarkers of dementia and neurodegeneration with SNI. In addition, we assessed potentially moderating effects on that association conferred by genes and viruses that have been implicated in dementia risk or protection.

### Apolipoprotein E (ApoE)

With regard to genetic influences, it is well-established that individual variation in ApoE influences risk of Alzheimer’s dementia (AD). Specifically, the presence of ApoE2 is associated with decreased risk of AD whereas ApoE4 increases AD risk^23–26^. Furthermore, ApoE4 has been linked to poorer cognitive performance in cognitively unimpaired individuals^27^. Finally, ApoE4 has been associated with altered brain function not only in AD^28^ but also in otherwise healthy adults^29^.

### Human leukocyte antigen (HLA)

In contrast to detrimental effects of ApoE4 on brain function, certain HLA alleles have been associated with protective effects. Specifically, DRB1*13 alleles (DRB1*13:01, DRB1*13:02) have been associated with protection against age-related brain atrophy and neural network variability (i.e., “noise”) even in the presence of ApoE4^30–32^. Various HLA alleles have been identified as a risk or protective factors for several human diseases^33^ including dementia^34–38^. HLA genes code for cell-surface proteins that are involved in the adaptive immune system response to foreign antigens such as viruses^39^; thus, the beneficial effects of certain HLA alleles are presumed to be related to an effective immune system response aimed at neutralizing virus (or other foreign) antigens that could otherwise contribute to disease^40,41^.

### Human viruses

Several viruses have been implicated in conditions affecting the brain including dementia^42^. In particular, human herpesviruses (HHV), several of which are neurotropic, have been implicated in AD^43–49^ and in impaired cognitive performance in healthy adults^50^. HHV infections are very common in the general population^51,52^ with seroprevalence estimates often > 90% for several HHVs^53^. We recently documented age-related increases in blood biomarkers of dementia in cognitively unimpaired women who were seropositive for HHVs compared to women who were seronegative, independently of ApoE4 presence^54^; that effect was strongest for HHV1 (herpes simplex virus 1, HSV1), HHV4 (Epstein-Barr Virus), and HHV6 (Roseola virus). In addition to HHVs, other common viruses, including human endogenous retroviruses (HERVs) and human papilloma virus (HPV) are associated with neurodegenerative disorders in observational and mechanistic studies^55–61^.

### This study

In this work, we integrated measures of brain function, blood biomarkers, genetics, and virus serology. Using a cohort of women who had not been diagnosed with dementia, we examined associations between blood biomarkers of dementia/neurodegeneration and neural network interactions, and evaluated the possible moderating effects of ApoE genotype, HLA-DRB1*13 alleles, and virus seropositivity.

## Results

### General

**Fig. 1:**
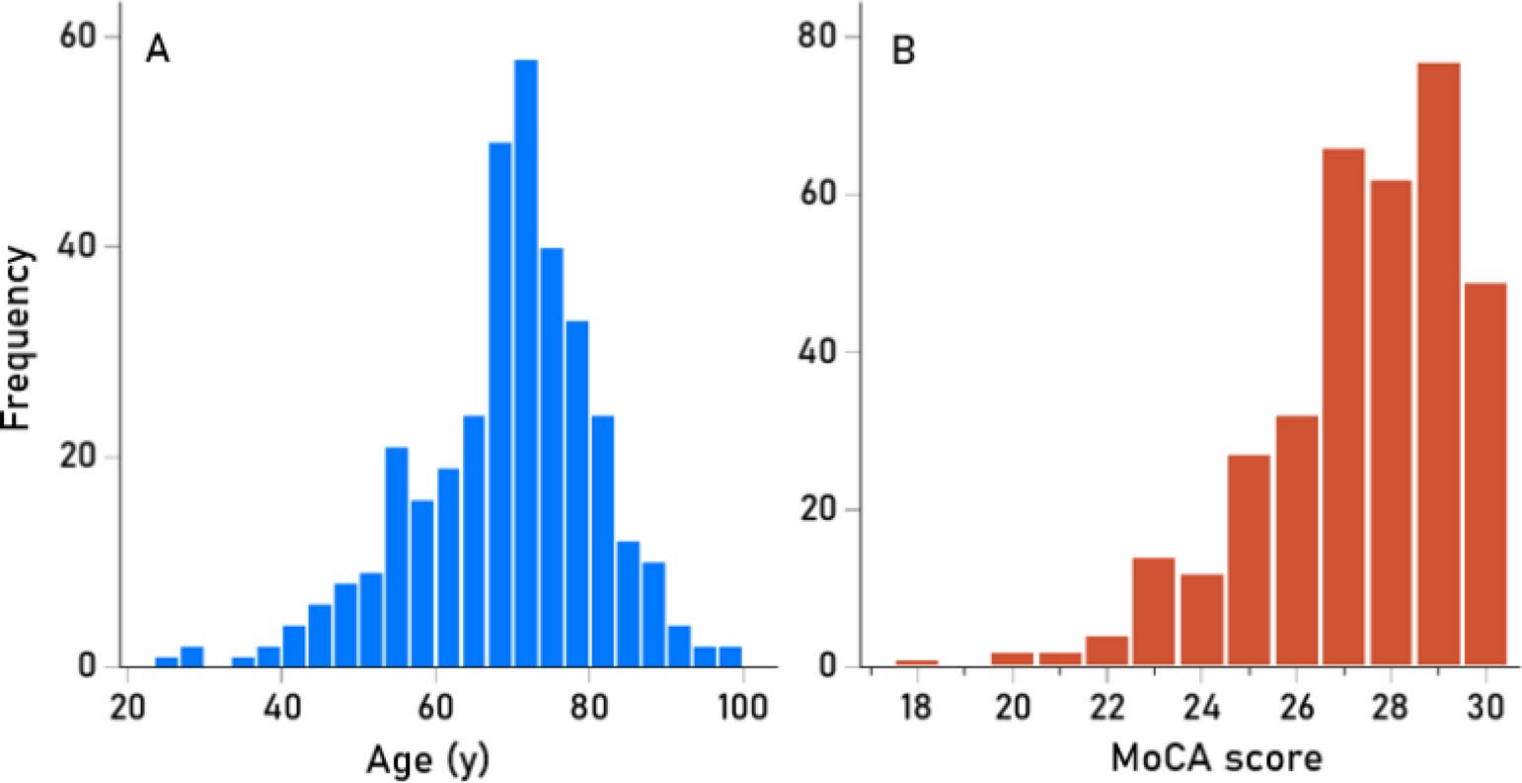
Frequency distributions of age (**A**) and MoCA scores (**B**). N = 348 acquisitions.

Descriptive statistics of age (Fig. 1A), Montreal Cognitive Assessment (MoCA) scores (Fig. 1B), seven biomarker measurements, and synchronous neural interactions (SNI) are given in Table 1. The ApoE distribution is presented in Table 2. With respect to HLA, we determined the presence/absence of HLA DRB1*13:01 and DRB1*13:02 for the 175 participants (Table 3). The seropositivity rates across the 348 acquisitions varied among the viruses tested (Table 4). HHV3 had a 100% seropositivity rate, so no further analyses were conducted for this virus.

**Table 1:** Descriptive statistics for the biomarkers used.

	Mean	SEM	Median	N
Age (y)	69.2	0.63	70.7	348
MoCA	27.4	0.11	28.0	348
Aβ40	53.4	2.75	46.3	348
Aβ42	8.33	0.35	7.78	348
Aβ42/Αβ40	0.208	0.016	0.149	313
NFL	52.5	3.10	32.1	347
tTau	3.51	0.18	2.53	318
pTau181	0.441	0.030	0.250	348
pTau217	1.070	0.094	0.545	348
SNI	0.0367	0.000331	0.0354	348

Values for MoCA (includes education point) and Aβ42/Αβ40 are pure numbers. All other biomarker values are pg/ml determined in serum.

**Table 2:** ApoE genotypes of participants and ApoE2, ApoE4 groups.

ApoE	Ν	%		
22	1	0.6	ApoE2[ε2ε2, ε3ε2]	23
23	22	12.6		
24	3	1.7		
33	108	62.1		
34	34	19.5	ApoE4[ε4ε4, ε4ε3]	40
44	6	3.4		
Total	174	100%		

Ν = 172 participants for whom ApoE determinations were available. See text for details.

**Table 3:** Genotypes of HLA DRB1*13:01 and DRB1*13:02 (N = 175 participants).

	DRB1*13:01		DRB1*13:02	
	N	%	N	%
Absent	153	87.4	160	91.4
Present	22	12.6	15	8.6
Total	175	100%	175	100%

**Table 4:** Prevalence of seropositivity for the viruses investigated.

Virus strain		Total N	N seropositive	% seropositive
HHV1	HSV1	345	198	42.2
HHV2	HSV2	342	77	22.5
HHV4	EBV	346	333	95.7
HHV5	CMV	347	185	53.2
HHV6	6A, B	331	304	87.4
HERVK		348	238	68.4
HERVW		348	317	91.1
HPV	6/11/16/18/31/33/45/52/58	348	183	52.6

Numbers in the column total indicate the number of tests that gave unambiguous results in the ELISA.

### Association of biomarkers with SNI

The possible association of the various biomarkers with the MEG measure SNI was evaluated using linear mixed-effects models [LMEM; see “Methods” section] where Subjects were the participants (coded as nominal variable), Repeated were the visits (coded as consecutive integers starting with 0), Random command included an intercept, SNI was the dependent variable, and age, Aβ40, Aβ42, Aβ42/40, NFL, Total Tau, pTau181, and pTau217 were covariates. The results are shown in Table 5 where it can be seen that only pTau217 had a significant, positive effect on SNI (*P* = 0.0027). Therefore, subsequent analyses were carried out only for pTau217.

**Table 5:** Results of biomarker-SNI associations.

Biomarker	*P* value	Biomarker-SNI association
Age	0.154 (NS)	
Aβ40	0.335 (NS)	
Aβ42	0.128 (NS)	
Aβ42/40	0.776 (NS)	
NFL	0.074 (NS)	
Total Tau	0.577 (NS)	
pTau181	0.376 (NS)	
pTau217	0.0027	Positive

LMEM analysis. *P* values refer to the testing the statistical significance of biomarker-SNI association (controlling for age).

### Effect of ApoE genotype on pTau217-SNI association

This effect was evaluated by performing LMEM as above (Subjects, Repeated, Random intercept), where SNI was the dependent variable, ApoE2_4 was a fixed factor for ApoE2[ε2ε2, ε3ε2] and ApoE4[ε4ε4, ε4ε3] groups, coded as a binary fixed factor), age and pTau217 were covariates, and ApoE2_4 × pTau217 was an interaction term. No statistically significant effect was found for the ApoE2_4 × pTau217 term (*P* = 0.295) indicating that the null hypothesis that the SNI vs. pTau217 slopes did not differ between the two ApoE2_4 groups could not be rejected.

### Effect of HLA DRB1*13:01 and DRB1*13:02 alleles on pTau217-SNI association

This effect was evaluated by performing a LMEM analysis where SNI was the dependent variable, DRB1*13:01 was a fixed factor (coded as a binary fixed factor), age and pTau217 were covariates, and DRB1*13:01 × pTau217 was an interaction term, The results revealed a statistically significant effect of DRB1*13:01 × pTau217 interaction term (*P* = 0.002) indicating that the SNI vs. pTau217 parameters (slopes) were not parallel between the carriers and non-carriers of the DRB1*13:01 allele. These slopes were compared directly by performing two LMEM analyses, one for the DRB1*13:01(−) group and another for the DRB1*13:01(+) group. In these analyses, SNI was the dependent variable, and age and pTau217 were covariates. The slope in the DRB1*13:01(+) group was negative and significantly smaller than that in the DRB1*13:01(−) group (positive) (Z = 4.646, *P* < 0.00001, Paternoster test). These results indicate that the presence of DRB1*13:01 allele lowers the positive dependence of SNI on pTau217. In contrast, the presence of DRB1*13:02 did not have a statistically significant effect.

### Effect of viruses on pTau217-SNI association

The effect of seroprevalence (presence/absence of seropositivity) on pTau217-SNI association of each virus was evaluated in the same way described above for other factors. The results are shown in Table 6. It can be seen that only seropositivity for HHV1 and HERVK had a statistically significant effect, both increasing the dependence of SNI on pTau217. Both HHV1 and HERVK effects were absent in carriers of DRB1*13:01 (Table 7). DRB1:13:02 did not have statistically significant effect. Notably, DRB1*13:01 showed strong predicted bindings (IC_50_ < 50 nM) to HHV1 and HERVK (best predicted binding affinity 22.3 and 10.0 nM, respectively) (Table 8).

**Table 6:** Effect of virus seropositivity on pTau217-SNI association.

	Interaction in LMEM	Comparison of slopes (Paternoster test)
HHV1	*P* = 0.008	*P* = 0.0027 (Virus present > Virus absent)
HHV2	NS	
HHV4	NS	
HHV5	NS	
HHV6/7	NS	
HERVK	*P* = 0.006	*P* = 0.0071 (Virus present > Virus absent)
HERVW	NS	
HPV	NS

**Table 7:** Effect of HHV1 and HERVK seropositivity on pTau217-SNI association in the absence/presence of DRB1*13:01.

		Interaction in LMEM	
		DRB1*13:01	
		Absent	Present
Virus	HHV1	*P* = 0.037(+)	NS
	HERVK	*P* < 0.001(+)	NS

**Table 8:** Viruses tested for predicted binding affinity.

Virus	Protein description	Uniprot ID	AA	N epitopes
HHV1	Envelope glycoprotein D	Q69091	394	380
HERVK	10 Pol protein	P10266	1014	1000

### MoCA

MoCA is an estimate of overall cognitive function. Here we evaluated the effects on MoCA of age, SNI, all 7 biomarkers, and DRB1*13:01 and DRB1*13:02 using LMEM analysis, where the MoCA score was the dependent variable, as described below.

#### Effect of age

In this LMEM analysis age was the sole covariate. We found a highly statistically significant negative effect of age on MoCA (*P* < 0.001), i.e. MoCA score decreased with age. In addition, in the same model, we evaluated the effect of individual biomarkers on MoCA, controlling for age. Of the 7 biomarkers, only pTau217 had a highly significant negative effect on MoCA (*P* < 0.001).

#### Effect of pTau217-SNI

In this LMEM analysis age, SNI and pTau217 were covariates and pTau217-SNI was an interaction term. We found a highly statistically significant negative effect of the pTau217-SNI interaction on MoCA (*P* < 0.001).

#### Effects of DRB1*13:01 and DRB1*13:02

The analysis above was performed for the absence/presence of DRB1*13:01 and, similarly, for DRB1*13:02. In the absence of either of these two HLA alleles, the pTau217-SNI association was associated with a highly significant negative effect on MoCA (*P* < 0.001 for DRB1*13:01 and *P* = 0.004 for DRB1*13:02). In contrast, no such significant association was found in carriers of either allele (*P* = 0.232 for DRB1*13:01 and *P* = 0.073 for DRB1*13:02).

## Discussion

### pTau217 and brain function

Despite individual differences in brain anatomy, synchronous neural interactions are remarkably consistent across individuals^3^ such that deviations are informative about brain health^4^. Here we show that brain network function is strongly associated with serum pTau217. We have previously shown that decrements in cognitive functioning are associated with increased neural network correlations which are thought to constrain information processing by reducing neural network flexibility^11^. Here, in an extension of those findings, we documented that cognitive performance declined as the pTau217-SNI association increased. The strong association of pTau217 with SNI was particularly robust in the presence of HHV1 or HERVK seropositivity. Remarkably, the pTau217-SNI association and its effect on MoCA were eliminated in the presence of HLA-DRB1*13:01, which is characterized by high-affinity binding to the viruses examined. These findings underscore the association of serum pTau217 on brain function and provide additional support for robust protective effects of DRB1*13:01, highlighting the central role of HLA-mediated adaptive immune responses in maintaining brain health.

Of the seven biomarkers evaluated in the present study, only pTau217 was significantly associated with brain functioning. Several prior studies have documented the sensitivity of pTau217. For example, pTau217 outperforms other biomarkers in diagnosis of AD, even at early stages of the disease, and in distinguishing AD from other neurodegenerative disorders^62–65^. Furthermore, blood pTau, and particularly pTau217, has been associated with longitudinal cognitive decline in a sample of non-demented individuals^66^, and has been shown to predict AD diagnosis when combined with ApoE and cognitive performance^67^. The mechanisms underlying the role of pTau217 on neurodegeneration are still under investigation; however, evidence suggests that toxic gain-of-function resulting from trans-synaptic spread of misfolded tau proteins as well as reduced clearance of pathogenic tau, neuroinflammation, and interaction with other proteins (e.g., amyloid beta) likely contribute to pathology, including neuronal dysfunction and synaptic degeneration^68,69^. Indeed, tau has been linked to numerous synaptic functions in healthy brains including regulation of synaptic mitochondria and neurotransmitter receptors as well as directly interacting with proteins and post-synaptic signaling complexes, any of which may be impaired by tau alterations including phosphorylation^70^. Notably, since MEG measures synaptic activity^1,2^ it may be optimally equipped to capture pTau217-associated synaptic degeneration. It is worth noting that the effects of pTau217 on brain function documented here were independent of ApoE. While some evidence suggests that pTau is more prone to drive trans-synaptic progression of tau pathology and tau-mediated synaptic disruption in ApoE4 carriers^71^, ApoE4-associated AD risk is variable^72^ and moderated by multiple factors that influence brain health and cognitive function^32,34,73–75^.

### Effect of virus seropositivity on pTau217 association with SNI

Causal connections between microbial infection and neurodegeneration, particularly AD, have been investigated for decades^76^, with several HHVs and other infectious agents implicated in cognitive decline and AD^50,77,78^. We recently reported age-related increase in blood biomarkers of AD in HHV seropositive individuals^54^. Here we found that history of infection with HHV1 or HERVK, both of which have been implicated in AD and neurodegenerative disease^43,44,56,57,79^, increase pTau217-SNI associations, controlling for age. These findings add to the extensive body of research linking viruses to cognitive decline and highlight the influence of virus infection on blood biomarkers of AD and brain function. Many viruses are neurotropic and/or capable of entering the brain via peripheral nerves or the bloodstream^80,81^, resulting in neuroinflammation^77^. Moreover, both tau and HHVs induce activation of HERVs^81^ which are increasingly recognized for their involvement in the pathogenesis of many brain disorders^55^; however, morbidity and mortality associated with HERVK is partially moderated by HLA^41,46,82^.

### HLA DRB1*13:01 reduces pTau217 association with brain function

Previous studies have documented protective effects of DRB1*13 alleles on brain atrophy, neuronal dysfunction, and various conditions affecting the brain^30–37^. In the present study, we found that the significant association between pTau217 and brain function was eliminated in DRB1*13:01 carriers; notably, this protective effect persisted even in the presence of virus seropositivity. Since HLA is instrumental in facilitating the human immune system response to viral antigens, the protective effects observed here are presumably due to enhanced immune system response conferred by DRB1*13:01. Indeed, DRB1*13:01 was found to bind with strong affinity to both HHV1 and HERVK, suggesting ability to eliminate and/or neutralize these viruses that might otherwise contribute to disease. The protective effect of DRB1*13:01 on pTau217-SNI association is in line with a proposed role of HLA in dementia prevention^41^. We proposed that HLA alleles capable of effectively binding viral or microbial antigens are likely to promote health. In contrast, insufficient HLA-antigen binding results in antigen persistence and neuronal damage^40^. One role of ApoE is neuronal repair, particularly in the case of ApoE2 and ApoE3; however, ApoE4 expression is associated with neurotoxic fragments^83^. Thus, synthesis of ApoE4 resulting from antigen persistence is likely to contribute to neurodegeneration. From this perspective, HLA exerts a primary influence on viral contributions to neurodegeneration.

## Summary, considerations, and limitations

Taken together, the present findings suggest that serum tau phosphorylation is significantly associated with neural dysfunction in non-demented women, particularly in those with history of certain viral infections who are lacking protective HLA alleles. Most participants in this sample were cognitively normal based on MoCA, a well-validated cognitive screening tool. Even so, blood biomarkers of dementia were detectable, and, in the case of pTau217, significantly associated with neuronal functioning. Given the negative association of pTau217-SNI with cognitive performance, we anticipate that these effects may be even more pronounced in samples of cognitively impaired individuals, although this remains to be investigated. Importantly, the protective effects documented here for DRB1*13:01 do not exclude contributions from other HLA alleles. HLA is highly polymorphic, and several variants have been implicated in brain function^32^ and AD risk^34–38^. In addition, the present study was limited to a subset of viruses; however, numerous other viruses and bacteria have been implicated in neurodegeneration and dementia. Finally, the study exclusively focused on women; in light of sex differences in dementia and immune responses^84,85^, it remains to be seen whether the current findings extend to men. Future studies evaluating sex differences regarding the influence of viruses and genetics on blood biomarkers and neuronal function are warranted.

## Materials and methods

### Participants

A total of 175 women participated in the study as paid volunteers. The data were obtained as part of an ongoing longitudinal study involving annual data acquisition; consequently, the number of annual visits varied for participants, for a total of 348 visits. Women were excluded from the study if they, at the time of consent, had been diagnosed at any point in their lifetime with a neurological disorder, any autoimmune disorder associated with neurocognitive dysfunction (e.g., systemic lupus erythematous, rheumatoid arthritis), any major medical condition affecting brain function (e.g., brain cancer, head injury with cognitive sequelae), serious psychiatric diagnoses (e.g., bipolar disorder, schizophrenia, any history of psychiatric hospitalization), or any recent/current medication or treatment known to affect brain function (e.g., radiation, chemotherapy). Finally, with respect to education, most participants were well educated: 7% had completed high school or equivalent, 17% had completed 3 years of college or less, and 76% of the had completed 4 years of college or more. Written informed consent was obtained from study participants. The institutional review board and relevant committees of the Minneapolis VA Health Care System approved the study protocol which was performed in accordance with the Declaration of Helsinki.

### Cognitive assessment

MoCA^86^ was administered to screen participants for cognitive impairment. MoCA assesses several domains of cognitive function including executive function, memory, language, and abstract reasoning, among others. Scores for each domain were added to reflect a total score ranging from 0 to 30 (without the education point).

### ApoE genotyping

Determination of ApoE genotype was performed as follows for 174/175 participants (ApoE could not be determined in one participant due to technical reasons). DNA samples were genotyped using PCR amplification followed by restriction enzyme digestion^87^. Each amplification reaction contained PCR buffer with 15 mmol/L MgCl 2 nanograms of of genomic DNA, 20 pmol ApoE forward (5N TAA GCT TGG CAC GGC TGT CCA AGG A 3N) and reverse (5N ATA AAT ATA AAA TAT AAA TAA CAG AAT TCG CCC CGG CCT GGT ACA C 3N) primers, 1.25 mmol/L of each deoxynucleotide triphosphate, 10% dimethylsulfoxide, and 0.25 μL AmpliTaq Gold DNA polymerase (Thermo Fisher Scientific, Waltham MA, USA). Reaction conditions in a thermocycler included an initial denaturing period of 3 min at 95 C, 1 min at 60 C, and 2 min at 72 C; followed by 32 cycles of 1 min at 95 C, 1 min at 60 C, and 2 min at 72 C; and a final extension of 1 min at 95 C, 1 min at 60 C, and 3 min at 72 C. PCR products were digested with HhaI and separated on a 4% Agarose gel which was stained with Ethidium Bromide. Known ApoE isoform standards were included in the analysis.

### Determination of blood biomarkers in serum

We determined the serum levels of the following dementia-related biomarkers: Aβ40, Aβ42, Neurofilament light (NFL), total Tau (tTau), phosphorylated Tau 181 (pTau181), and phosphorylated Tau217 (PtAU217), as follows. Αβ40 and Αβ42 were determined using the FUJIFILM Wako ELISA kits (Catalog # 296-64401 and 298-64601). NFL and tTau were determined on the Ella Automated Immunoassay System (ProteinSimple, part of Bio-Techne) using the Simple Plex Human Total Tau (Catalog # SPCKC-PS-009562) and NFL (Catalog # SPCKB-PS-002448) cartridges. pTau181 and pTau217 were determined on a MESO SECTOR S 600MM using the S-PLEX Human Tau kits (Meso Scale Discovery, Catalog # K151AGMS and K151APFS). Assays were performed and values calculated following the manufacturers’ instructions. Serum samples were diluted 1:2 by the recommended diluent.

### HLA genotyping

DNA isolation was carried out from whole blood or saliva samples using commercially available kits (blood: ArchivePure cat# 2300730 from 5Prime distributed by Fisher Scientific or VWR; saliva: Oragene-Discover cat.OGR-500 coupled with prepIT purifier reagent cat.PT-L2P/DNA Genotek Inc. Ottawa, ON, Canada). The purified DNA samples were sent to HistoGenetics (http://www.histogenetics.com/) for high-resolution HLA Sequence-based Typing (SBT; details are given in https://bioinformatics.bethematchclinical.org/HLA-Resources/HLA-Typing/High-Resolution-Typing-Procedures/ and https://bioinformatics.bethematchclinical.org/WorkArea/DownloadAsset.aspx?id=6482). Their sequencing DNA templates are produced by locus- and group-specific amplifications that include exon 2 and 3 for Class I (A, B, C) and exon 2 for Class II (DRB1, DRB3/4/5, DQB1, and DPB1) and reported as Antigen Recognition Site (ARS) alleles as per ASHI recommendation^88^.

### Virus seropositivity assays

We determined the seroprevalence of IgG antibodies against Human Herpes Virus 1(HHV1), HHV2, HHV3, HHV4, HHV5 and HHV6 using commercially available enzyme-linked immunosorbent assay (ELISA) kits; we could not obtain reliable results from kits for HHV7 and HHV8. The ELISA tests were performed as per the manufacturer’s instructions and recommendations. Serum samples were processed using a mini-automated 5-in-1 workstation (Crocodile cat. 84024-01; Berthold Technologies, Oak Ridge, TN, USA). The workstation includes the ELISA microtiter plate reader which was read at dual wavelengths for absorbance at 450 nm and 620 nm as reference wavelengths. Details of the virus-specific ELISA kits are as follows: (a) HHV1: Human Anti-Herpes simplex virus Type 1 IgG ELISA Kit (HSV1), Abcam Inc., Boston, MA USA, cat. ab 108737; (b) HHV2: Human Anti-Herpes simplex virus Type 2 IgG ELISA Kit (HSV2) Abcam cat. ab 108739; (c) HHV3: Human Anti-Varicella-Zoster Virus IgG ELISA Kit (VZV) Abcam cat. ab 108782; (d) HHV4: Human Anti-Epstein Barr virus IgG ELISA Kit (EBV-VCA) Abcam cat. ab 108730; (e) Human Anti-Cytomegalovirus IgG ELISA Kit (CMV) Abcam cat. ab 108724; (f) Human Herpesvirus 6 IgG ELISA Kit cat. KA1457, Abnova, Taiwan. Ambiguous readings were not used in the analyses. (g) ERVK6 ELISA Aviva Systems Biology kit OKEH08186 at 1:4. (h) Syncytin-1/ERVW-1 Abbexa kit abx522100 at 1:5. (i) HPVL1 ELISA: Anti-HPV L1 IgG (types 6/11/16/18/31/33/45/52/58) Alpha Diagnostic International kit 550-500-PHG at 1:200, All assays were performed according to the respective manufacturers’ instructions.

### In silico determination of predicted best binding affinities of DRB1*13:01

**Fig. 2:**
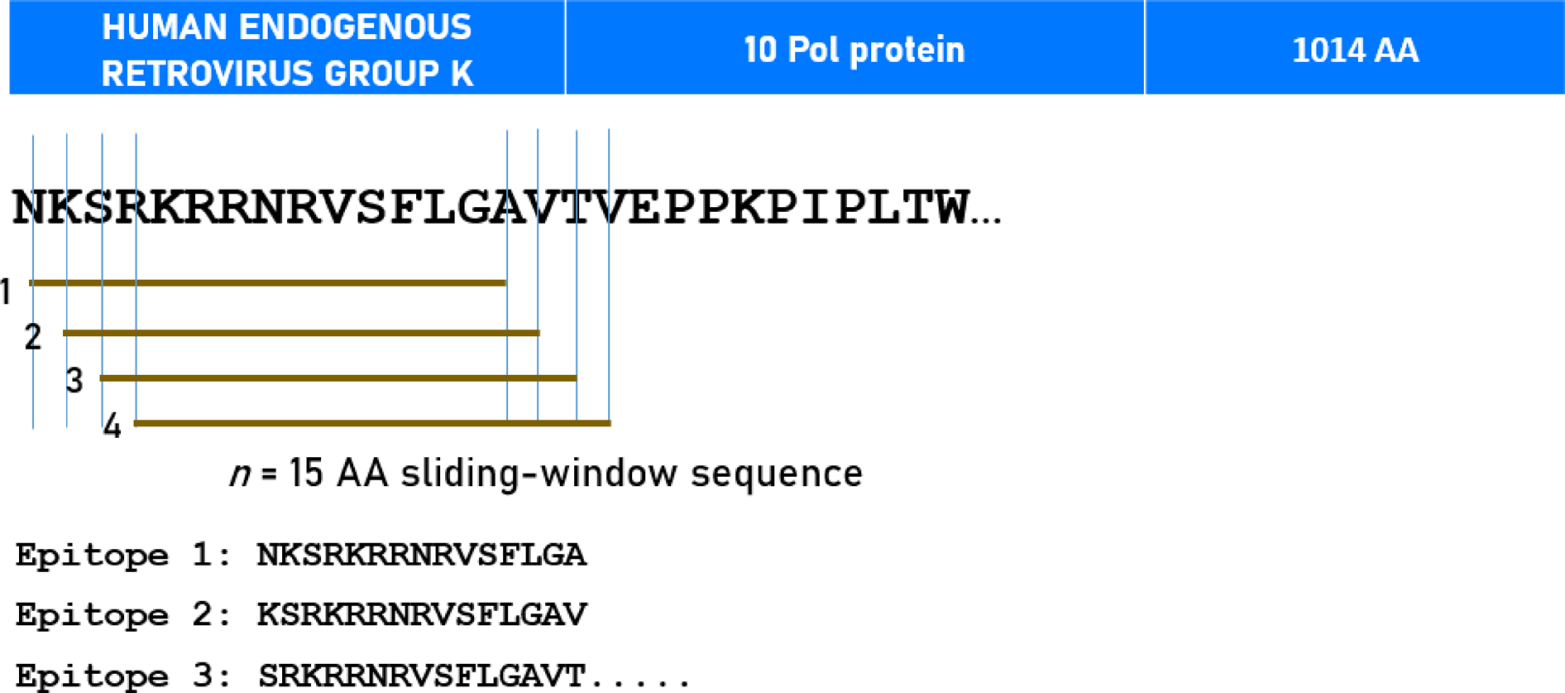
Schematic diagram to illustrate the sliding window approach for in silico testing epitope binding affinities to DRB1*13:01. See text for details.

We estimated in silico the predicted best binding affinity of DRB1*13:01 to the viruses that were associated with biomarker-brain associations (HHV1 and HERVK) using the Immune Epitope Database (IEDB; http://tools.iedb.org/mhci/) NetMHCpan (ver. 4.1 BA) tool^89^. We used the sliding window approach^90,91^ to test exhaustively all possible linear 15*-mer* epitopes of the virus proteins analyzed (Table S1, Supplementary Material); the epitope length of 15 amino acids is optimal for HLA-II molecule binding^92–94^. The method is illustrated in Fig. 2 for HERVK. For each pair of peptide-HLA molecule (pHLA-II) tested, this tool gave as an output the IC_50_ of the predicted binding affinity; the smaller the IC_50_, the stronger the binding affinity. Given a protein of *N* amino acid length and an epitope length of 15 AA, there were *N-15* + 1 IC_50_ values. The predicted *best* binding affinity (PBBA) for each pHLA-II pair was the minimum IC_50_ value of all epitopes tested for the pair. An IC_50_ value of IC_50_ < 50 nm is regarded strong^95^ (“hit”). The number of epitopes tested for each virus is given in Table S1.

### Magnetoencephalography (MEG)

All participants underwent a MEG scan. As described previously^4^, participants lay supine within the electromagnetically shielded chamber and fixated their eyes on a spot 65 cm in front of them, for 60 s. MEG data were acquired using a 248-channel axial gradiometer system (Magnes 3600WH, 4-D Neuroimaging, San Diego, CA), band-filtered between 0.1 and 400 Hz, and sampled at 1017.25 Hz, corresponding to a sampling interval of 0.983 ms. Data with artifacts (e.g. from excessive subject motion) were eliminated from further analysis. MEG records were visually screened and rejected if artifacts were present (e.g. from excessive subject motion, eye movements, blinking, or environmental noise).

Processing of the raw MEG series was performed using programs in Python^96^. Single trial MEG time series from all sensors underwent ‘prewhitening’^97^ using a (50, 1, 3) Auto Regressive Integrated Moving Average (ARIMA) model to obtain innovations (i.e. residuals)^96^. All possible pairwise zero-lag crosscorrelations, $r$ (Synchronous Neural Interactions, SNI; N = $\frac{248 \times 247}{2}$ = 30,628 sensor pairs) were computed between the prewhitened MEG time series of each MEG scan. Crosscorrelations were transformed to $r_{z}$ using Fisher’s^98^ z-transformation to normalize their distribution:

1 $$r_{z} = {\mathrm{arctanh}}\left( r \right) = \frac{1}{2}\ln \left( {\frac{1 + r}{{1 - r}}} \right)$$

Finally, since we focused on the strength of neural interactions, irrespective of their sign, we used the absolute value of $r_{z}$ for further analyses:

2 $${\mathrm{SNI}} = \left| {r_{z} } \right|$$

### Statistical analyses

Statistical analyses were performed using the IBM-SPSS statistical package (version 30) using standard statistical methods. Since data at different visits were acquired from the same participants in several cases, a linear mixed-effects models (LMEM) analysis was performed using the mixed models linear procedure in SPSS, where Subjects were the participants and Visits were the Repeated variable (expressed as consecutive integers with the first one set to zero), Random command with intercept; SNI was the dependent variable, various binary groups (ApoE, HLA) were fixed factors, and age and biomarkers were covariates. Additionally, the effect of fixed factors (ApoE, DRB1 alleles) on the pTau217-SNI association was assessed by including a Fixed Factor main effect and a Fixed Factor × pTau217 interaction term to test the null hypothesis that the SNI vs. pTau217 parameters (slopes) are not parallel. If this interaction term was statistically significant, rejecting the null hypothesis above, the two slopes were compared directly using the Paternoster test^99^:

3 $$Z = \frac{{b_{1} - b_{2} }}{{\sqrt {SEb_{1}^{2} + SEb_{2}^{2} } }}$$

where $b_{1}$ and $b_{2}$ are LMEM parameters estimates (slopes), SE are their standard errors, and Z is the normal deviate. All *P* values reported are 2-sided, $a = 0.05$.

## Supplementary Information

Below is the link to the electronic supplementary material.

Supplementary Material 1

## Data Availability

The data that support the findings of this study are available from the corresponding author upon reasonable request.

## References

[1] Hamalainen, M. & Hari, R. Magnetoencephalographic characterization of dynamic brain activation: Basic principles and methods of data collection and source analysis. In *Brain Mapping: The Methods* 2nd edn (eds Toga, A. W. & Mazziotta, J. C.) 227–253 (Academic Press, 2002).

[2] Okada, Y. Neurogenesis of evoked magnetic fields. In *Biomagnetism: An Interdisciplinary Approach* (eds Williamson, S. H. et al.) 300–408 (Plenum Press, 1982).

[3] Langheim, F. J., Leuthold, A. C. & Georgopoulos, A. P. Synchronous dynamic brain networks revealed by magnetoencephalography. *Proc. Natl. Acad. Sci. U.S.A.* **103**, 455–459. 10.1073/pnas.0509623102 (2006). 16387850 10.1073/pnas.0509623102 PMC1324790

[4] Georgopoulos, A. P. et al. Synchronous neural interactions assessed by magnetoencephalography: A functional biomarker for brain disorders. *J. Neural Eng.* **4**, 349–355. 10.1088/1741-2560/4/4/001 (2007). 18057502 10.1088/1741-2560/4/4/001

[5] Georgopoulos, A. P. et al. Gulf War illness (GWI) as a neuroimmune disease. *Exp. Brain Res.* **235**, 3217–3225. 10.1007/s00221-017-5050-0 (2017). 28762055 10.1007/s00221-017-5050-0

[6] Georgopoulos, A. P. et al. The synchronous neural interactions test as a functional neuromarker for post-traumatic stress disorder (PTSD): A robust classification method based on the bootstrap. *J. Neural Eng.* **7**, 016011. 10.1088/1741-2560/7/1/016011 (2010). 10.1088/1741-2560/7/1/016011 20086271

[7] James, L. M., Leuthold, A. F. & Georgopoulos, A. P. MEG neural signature of sexual trauma in women veterans with PTSD. *Exp. Brain Res.* **240**, 2135–2142. 10.1007/s00221-022-06405-8 (2022). 35786746 10.1007/s00221-022-06405-8

[8] Voytek, B. & Knight, R. T. Dynamic network communication as a unifying neural basis for cognition, development, aging, and disease. *Biol. Psychiatry* **77**, 1089–1097. 10.1016/j.biopsych.2015.04.016 (2015). 26005114 10.1016/j.biopsych.2015.04.016 PMC4443259

[9] Verdoorn, T. A. et al. Evaluation and tracking of Alzheimer’s disease severity using resting-state magnetoencephalography. *J. Alzheimers Dis.* **26**, 239–255. 10.3233/JAD-2011-0056 (2011). 21971464 10.3233/JAD-2011-0056

[10] Mandal, P. K., Banerjee, A., Tripathi, M. & Sharma, A. A comprehensive review of magnetoencephalography (MEG) studies for brain functionality in healthy aging and Alzheimer’s disease (AD). *Front. Comput. Neurosci.* **12**, 60. 10.3389/fncom.2018.00060.12 (2018). 30190674 10.3389/fncom.2018.00060 PMC6115612

[11] James, L. M., Leuthold, A. C., Dolan, S. & Georgopoulos, A. P. Dependence of cognitive ability on synchronous neural interactions determined by magnetoencephalography. *J. Neurophysiol.* **129**, 963–967. 10.1152/jn.00077.2023 (2023). 37010135 10.1152/jn.00077.2023 PMC10110728

[12] Olshausen, B. A. & Field, D. J. Sparse coding of sensory inputs. *Curr. Opin. Neurobiol.* **14**, 481–487. 10.1016/j.conb.2004.07.007.21 (2004). 15321069 10.1016/j.conb.2004.07.007

[13] Vinje, W. E. & Gallant, J. L. Sparse coding and decorrelation in primary visual cortex during natural vision. *Science* **287**, 1273–1276. 10.1126/science.287.5456.1273.22 (2000). 10678835 10.1126/science.287.5456.1273

[14] Mielke, M. M. & Fowler, N. R. Alzheimer disease blood biomarkers: Considerations for population-level use. *Nat. Rev. Neurol.* **20**, 495–504. 10.1038/s41582-024-00989-1 (2024). 38862788 10.1038/s41582-024-00989-1 PMC11347965

[15] Hansson, O., Blennow, K., Zetterberg, H. & Dage, J. Blood biomarkers for Alzheimer’s disease in clinical practice and trials. *Nat. Aging* **3**, 506–519. 10.1038/s43587-023-00403-3 (2023). 37202517 10.1038/s43587-023-00403-3 PMC10979350

[16] Barthélemy, N. R. et al. Highly accurate blood test for Alzheimer’s disease is similar or superior to clinical cerebrospinal fluid tests. *Nat. Med.* **30**, 1085–1095. 10.1038/s41591-024-02869-z (2024). 38382645 10.1038/s41591-024-02869-z PMC11031399

[17] Sarkar, S. et al. Correlation between cognitive impairment and peripheral biomarkers-Significance of phosphorylated tau and amyloid-β in Alzheimer’s disease: A new insight. *Curr. Psychiatry Res. Rev.* **21**, 1–25. 10.2174/0126660822329981241007105405 (2025).

[18] Dubois, B. et al. Biomarkers in Alzheimer’s disease: Role in early and differential diagnosis and recognition of atypical variants. *Alzheimer Res. Ther.* **15**, 175. 10.1186/s13195-023-01314-6 (2023). 10.1186/s13195-023-01314-6 PMC10571241 37833762

[19] Mattsson, N., Cullen, N. C., Andreasson, U., Zetterberg, H. & Blennow, K. Association between longitudinal plasma neurofilament light and neurodegeneration in patients with Alzheimer disease. *JAMA Neurol.* **76**, 791–799. 10.1001/jamaneurol.2019.0765 (2019). 31009028 10.1001/jamaneurol.2019.0765 PMC6583067

[20] Marks, J. D. et al. Comparison of plasma neurofilament light and total tau as neurodegeneration markers: Associations with cognitive and neuroimaging outcomes. *Alzheimer’s Res. Ther.* **13**, 199. 10.1186/s13195-021-00944-y (2021). 34906229 10.1186/s13195-021-00944-y PMC8672619

[21] Jia, J. et al. Biomarker changes during 20 years preceding Alzheimer’s disease. *NEJM* **390**, 712–722. 10.1056/NEJMoa2310168 (2024). 38381674 10.1056/NEJMoa2310168

[22] Lu, Y. et al. Changes in Alzheimer disease blood biomarkers and associations with incident all-cause dementia. *JAMA* **332**, 1258–1269. 10.1001/jama.2024.6619 (2024). 39068543 10.1001/jama.2024.6619 PMC11284635

[23] Strittmatter, W. J. et al. Apolipoprotein E: High-avidity binding to beta-amyloid and increased frequency of type 4 allele in late onset familial Alzheimer disease. *Proc. Natl. Acad. Sci. U.S.A.* **90**, 1977–1981. 10.1073/pnas.90.5.1977 (1993). 8446617 10.1073/pnas.90.5.1977 PMC46003

[24] Corder, E. H. et al. Gene dose of apolipoprotein E type 4 allele and the risk of Alzheimer’s disease in late onset families. *Science* **261**, 921–923. 10.1126/science.8346443 (1993). 8346443 10.1126/science.8346443

[25] Liu, C. C. et al. Apolipoprotein E and Alzheimer disease: Risk, mechanisms and therapy. *Nat. Rev. Neurol.* **9**, 106–118. 10.1038/nrneurol.2012.263 (2013). 23296339 10.1038/nrneurol.2012.263 PMC3726719

[26] Serrano-Pozo, A., Das, S. & Hyman, B. T. APOE and Alzheimer’s disease: Advances in genetics, pathophysiology, and therapeutic approaches. *Lancet. Neurol.* **20**(1), 68–80. 10.1016/S1474-4422(20)30412-9 (2021). 33340485 10.1016/S1474-4422(20)30412-9 PMC8096522

[27] Wisdom, N. M., Callahan, J. L. & Hawkins, K. A. The effects of apolipoprotein E on non impaired cognitive functioning: A meta-analysis. *Neurobiol. Aging* **32**, 63–74. 10.1016/j.neurobiolaging.2009.02.003 (2011). 19285755 10.1016/j.neurobiolaging.2009.02.003

[28] Canuet, L. et al. Resting-state network disruption and APOE ge notype in Alzheimer’s disease: A lagged functional connectivity study. *PLoS ONE* **7**, e46289. 10.1371/journal.pone.0046289 (2012). 23050006 10.1371/journal.pone.0046289 PMC3457973

[29] Leuthold, A. C., Mahan, M. Y., Stanwyck, J. J., Georgopoulos, A. & Georgopoulos, A. P. The number of cysteine residues per mole in apolipoprotein E affects systematically synchronous neural interactions in women’s healthy brains. *Exp. Brain Res.* **226**, 525–536. 10.1007/s00221-013-3464-x (2013). 23503772 10.1007/s00221-013-3464-x

[30] James, L. M. et al. Protective effect of human leukocyte antigen (HLA) allele DRB1*13:02 on age-related brain gray matter volume reduction in healthy women. *EBioMedicine* **29**, 31–37. 10.1016/j.ebiom.2018.02.005 (2018). 29452862 10.1016/j.ebiom.2018.02.005 PMC5925575

[31] James, L. M. et al. The effects of human leukocyte antigen DRB1*13 and apolipoprotein E on age-related variability of synchronous neural interactions in healthy women. *EBioMedicine* **35**, 288–294. 10.1016/j.ebiom.2018.08.026 (2018). 30139626 10.1016/j.ebiom.2018.08.026 PMC6161538

[32] James, L. M., Leuthold, A. C. & Georgopoulos, A. P. Human leukocyte antigen (HLA) modulates the dependence on age of the variability of synchronous neural interactions. *Neurosci. Insights* **18**, 1–8. 10.1177/26331055231159658 (2023). 10.1177/26331055231159658 PMC10037734 36969700

[33] Dendrou, C. A., Petersen, J., Rossjohn, J. & Fugger, L. HLA variation and disease. *Nat. Rev. Immunol.* **18**, 325–339. 10.1038/nri.2017.143 (2018). 29292391 10.1038/nri.2017.143

[34] Lambert, J. C. et al. Meta-analysis in more than 74,000 individuals identifies 11 new susceptibility loci for Alzheimer’s disease. *Nat. Genet.* **45**, 1452–1458. 10.1038/ng.2802 (2013). 24162737 10.1038/ng.2802 PMC3896259

[35] Steele, N. Z. et al. Fine-mapping of the human leukocyte antigen locus as a risk factor for Alzheimer disease: A case-control study. *PLoS Med.* **14**, e1002272. 10.1371/journal.pmed.1002272 (2017). 28350795 10.1371/journal.pmed.1002272 PMC5369701

[36] Wang, Z. X. et al. Genetic association of HLA gene variants with MRI brain structure in Alzheimer’s disease. *Mol. Neurobiol.* **54**, 3195–3204. 10.1007/s12035-016-9889-z (2017). 27056077 10.1007/s12035-016-9889-z

[37] James, L. M. & Georgopoulos, A. P. Immunogenetic epidemiology of dementia and Parkinson’s disease in 14 continental European countries: Shared human leukocyte antigen (HLA) profiles. *J. Immunol. Sci.* **5**, 16–26. 10.29245/2578-3009/2021/2.1209 (2021). 40370814 10.29245/2578-3009/2021/2.1209 PMC12077081

[38] James, L. M. & Georgopoulos, A. P. Tri-allelic human leukocyte antigen (HLA) protection against dementia. *J. Neurol. Neuromed.* **5**, 12–17. 10.29245/2572.942x/2019/1.1261 (2019). 10.29245/2572.942x/2019/1.1261 PMC12311969 40747291

[39] Meuer, S. C. et al. Surface structures involved in target recognition by human cytotoxic T lymphocytes. *Science* **218**, 471–473. 10.1126/science.6981845 (1982). 6981845 10.1126/science.6981845

[40] James, L. M. & Georgopoulos, A. P. Persistent antigens hypothesis: The human leukocyte antigen (HLA) connection. *J. Neurol. Neuromed.* **3**, 27–31. 10.29245/2572.942x/2018/6.1235 (2018). 10.29245/2572.942x/2018/6.1235 PMC12077249 40370508

[41] James, L. M. & Georgopoulos, A. P. Human leukocyte antigen as a key factor in preventing dementia and associated apolipoprotein E4 risk. *Front. Aging Neurosci.* **11**, 82. 10.3389/fnagi.2019.00082 (2019). 31031617 10.3389/fnagi.2019.00082 PMC6473084

[42] Mielcarska, M. B. & Rouse, B. T. Viruses and the brain—A relationship prone to trouble. *Viruses* **17**, 203. 10.3390/v17020203 (2025). 40006958 10.3390/v17020203 PMC11860391

[43] Itzhaki, R. F. Corroboration of a major role for herpes simplex virus type 1 in Alzheimer’s disease. *Front. Aging Neurosci.* **10**, e00324. 10.3389/fnagi.2018.00324 (2018). 10.3389/fnagi.2018.00324 PMC6202583 30405395

[44] Itzhaki, R. F. Infections, vaccinations, and risk of Alzheimer’s disease (AD)/dementia: Probable involvement of reactivated herpes simplex virus type 1. *Adv. Exp. Med. Biol.* **1423**, 279–280. 10.1007/978-3-031-31978-5_28 (2023). 37525055 10.1007/978-3-031-31978-5_28

[45] Cairns, D. M., Itzhaki, R. F. & Kaplan, D. L. Potential involvement of varicella zoster virus in Alzheimer’s disease via reactivation of quiescent herpes simplex virus type 1. *J. Alzheimers Dis.* **88**, 1189–1200. 10.3233/JAD-220287 (2022). 35754275 10.3233/JAD-220287

[46] James, L. M., Charonis, S. A. & Georgopoulos, A. P. Association of dementia human leukocyte antigen (HLA) profile with human herpes viruses 3 and 7: An in silico investigation. *J. Immunol. Sci.* **5**, 7–14. 10.29245/2578-3009/2021/3.1218 (2021). 40371217 10.29245/2578-3009/2021/3.1218 PMC12077050

[47] Charonis, S., James, L. M. & Georgopoulos, A. P. In silico assessment of binding affinities of three dementia-protective human leukocyte antigen (HLA) alleles to nine human herpes virus antigens. *Curr. Res. Transl. Med.* **68**, 211–216. 10.1016/j.retram.2020.06.002 (2020). 32624427 10.1016/j.retram.2020.06.002

[48] Readhead, B. et al. Multiscale analysis of independent Alzheimer’s cohorts finds disruption of molecular, genetic, and clinical networks by human herpesvirus. *Neuron* **99**, 64–82. 10.1016/j.neuron.2018.05.023 (2018). 29937276 10.1016/j.neuron.2018.05.023 PMC6551233

[49] Komaroff, A. L., Pellett, P. E. & Jacobson, S. Human herpesviruses 6A and 6B in brain diseases: Association versus causation. *Clin. Microbiol. Rev.* **34**, 10–128. 10.1128/CMR.00143-20 (2020). 10.1128/CMR.00143-20 PMC7667666 33177186

[50] Weidung, B. et al. Longitudinal effects of herpesviruses on multiple cognitive outcomes in healthy elderly adults. *J. Alzheimers Dis.* **94**, 751–762. 10.3233/JAD-221116 (2023). 37334589 10.3233/JAD-221116 PMC10357165

[51] Zuhair, M. et al. Estimation of the worldwide seroprevalence of cytomegalovirus: A systematic review and meta-analysis. *Rev. Med. Virol.* **29**, e2034. 10.1002/rmv.2034 (2019). 30706584 10.1002/rmv.2034

[52] Wald, A. & Corey, L. Persistence in the population: Epidemiology, transmission. In *Human Herpesviruses: Biology, Therapy, and Immunoprophylaxis, Chapter 36* (eds Arvin, A. et al.) (Cambridge University Press, 2007). 21348071

[53] Kuri, A. et al. Epidemiology of Epstein-Barr virus infection and infectious mononucleosis in the United Kingdom. *BMC Public Health* **20**, 912. 10.1186/s12889-020-09049-x (2020). 32532296 10.1186/s12889-020-09049-x PMC7291753

[54] James, L. M., Stratigopoulos, G. & Georgopoulos, A. P. Human herpes viruses are associated with steeper age-dependent increases of serum biomarkers for dementia in cognitively unimpaired women. *Sci. Rep.* **15**, 25475. 10.1038/s41598-025-10102-1 (2025). 40664837 10.1038/s41598-025-10102-1 PMC12263949

[55] Meyer, U. & Penner, I. K. Endogenous retroviruses in neurodevelopmental, psychotic and cognitive disorders. *Microbes Infect.*10.1016/j.micinf.2025.105479 (2025). 39914656 10.1016/j.micinf.2025.105479

[56] Adler, G. L., Le, K., Fu, Y. H. & Kim, W. S. Human endogenous retroviruses in neurodegenerative diseases. *Genes* **15**, 745. 10.3390/genes15060745 (2024). 38927681 10.3390/genes15060745 PMC11202925

[57] Tejeda, M. et al. DNA from multiple viral species is associated with Alzheimer’s disease risk. *Alzheimers Dement.* **20**, 253–265. 10.1002/alz.13414 (2024). 37578203 10.1002/alz.13414 PMC10840621

[58] Farrer, T. J., Moore, J. D., Chase, M., Gale, S. D. & Hedges, D. W. Infectious disease as a modifiable risk factor for dementia: A narrative review. *Pathogens* **13**, 974. 10.3390/pathogens13110974 (2024). 39599527 10.3390/pathogens13110974 PMC11597442

[59] Phan, K. et al. Pathological manifestation of human endogenous retrovirus K in frontotemporal dementia. *Commun. Med.* **1**, 60. 10.1038/s43856-021-00060-w (2021). 35083468 10.1038/s43856-021-00060-w PMC8788987

[60] Kozubek, P. et al. Human endogenous retroviruses and their putative role in pathogenesis of Alzheimer’s disease, inflammation, and senescence. *Biomedicines* **13**(1), 59. 10.3390/biomedicines13010059 (2025). 10.3390/biomedicines13010059 PMC11762123 39857643

[61] Oyouni, A. A. A. Human papillomavirus in cancer: Infection, disease transmission, and progress in vaccines. *J. Infect. Public Health* **16**, 626–631. 10.1016/j.jiph.2023.02.014 (2023). 36868166 10.1016/j.jiph.2023.02.014

[62] Barthélemy, N. R. et al. Cerebrospinal fluid phospho-tau T217 outperforms T181 as a biomarker for the differential diagnosis of Alzheimer’s disease and PET amyloid-positive patient identification. *Alzheimers Res. Ther.* **12**, 26. 10.1186/s13195-020-00596-4 (2020). 32183883 10.1186/s13195-020-00596-4 PMC7079453

[63] Janelidze, S. et al. Associations of plasma phospho-tau217 levels with tau positron emission tomography in early Alzheimer disease. *JAMA Neurol.* **78**, 149–156. 10.1001/jamaneurol.2020.4201 (2021). 33165506 10.1001/jamaneurol.2020.4201 PMC7653537

[64] Thijssen, E. H. et al. Plasma phosphorylated tau 217 and phosphorylated tau 181 as biomarkers in Alzheimer’s disease and frontotemporal lobar degeneration: A retrospective diagnostic performance study. *Lancet Neurol.* **20**, 739–752. 10.1016/s1474-4422(21)00214-3 (2021). 34418401 10.1016/S1474-4422(21)00214-3 PMC8711249

[65] Palmqvist, S. et al. Discriminative accuracy of plasma phospho-tau217 for Alzheimer disease vs other neurodegenerative disorders. *JAMA* **324**, 772–781. 10.1001/jama.2020.12134 (2020). 32722745 10.1001/jama.2020.12134 PMC7388060

[66] Pereira, J. B. et al. Plasma markers predict changes in amyloid, tau, atrophy and cognition in non-demented subjects. *Brain* **44**, 2826–2836. 10.1093/brain/awab163 (2021). 10.1093/brain/awab163 PMC8557344 34077494

[67] Palmqvist, S. et al. Prediction of future Alzheimer’s disease dementia using plasma phospho-tau combined with other accessible measures. *Nat. Med.* **27**, 1034–1042. 10.1038/s41591-021-01348-z (2021). 34031605 10.1038/s41591-021-01348-z

[68] Samudra, N., Lane-Donovan, C., VandeVrede, L. & Boxer, A. L. Tau pathology in neurodegenerative disease: Disease mechanisms and therapeutic avenues. *J. Clin. Investig.* **133**, e168553. 10.1172/JCI168553 (2023). 37317972 10.1172/JCI168553 PMC10266783

[69] Zhang, Y. et al. Tauopathies: New perspectives and challenges. *Mol. Neurodegener.* **17**, 28. 10.1186/s13024-022-00533-z (2022). 35392986 10.1186/s13024-022-00533-z PMC8991707

[70] Pooler, A. M., Noble, W. & Hanger, D. P. A role for tau at the synapse in Alzheimer’s disease pathogenesis. *Neuropharmacology* **76**, 1–8. 10.1016/j.neuropharm.2013.09.018 (2014). 24076336 10.1016/j.neuropharm.2013.09.018

[71] Thierry, M. et al. The influence of *APOE*^*ε4*^ on the pTau interactome in sporadic Alzheimer’s disease. *Acta Neuropathol.* **147**, 91. 10.1007/s00401-024-02744-8 (2024). 38772917 10.1007/s00401-024-02744-8 PMC11108952

[72] Patel, S. et al. Refining risk for Alzheimer’s disease among heterozygous APOEɛ4 carriers. *J. Alzheimers Dis.* **94**, 483–489. 10.3233/JAD-230156 (2023). 37334598 10.3233/JAD-230156

[73] Pearce, A. M., Marr, C., Dewar, M. & Gow, A. J. Apolipoprotein E genotype moderation of the association between physical activity and brain health. A systematic review and meta-analysis. *Front. Aging Neurosci.* **13**, 815439. 10.3389/fnagi.2021.815439 (2022). 35153725 10.3389/fnagi.2021.815439 PMC8833849

[74] Yassine, H. N. & Finch, C. E. *APOE* alleles and diet in brain aging and Alzheimer’s disease. *Front. Aging Neurosci.* **12**, 150. 10.3389/fnagi.2020.00150 (2020). 32587511 10.3389/fnagi.2020.00150 PMC7297981

[75] Angelopoulou, E., Paudel, Y. N., Papageorgiou, S. G. & Piperi, C. APOE genotype and Alzheimer’s disease: The influence of lifestyle and environmental factors. *ACS Chem. Neurosci.* **12**, 2749–2764. 10.1021/acschemneuro.1c00295 (2021). 34275270 10.1021/acschemneuro.1c00295

[76] Itzhaki, R. F. et al. Microbes and Alzheimer’s disease. *J. Alzheimers Dis.* **51**, 979–984. 10.3233/JAD-160152 (2016). 26967229 10.3233/JAD-160152 PMC5457904

[77] Ecarnot, F. et al. Dementia, infections and vaccines: 30 years of controversy. *Aging Clin. Exp. Res.* **35**, 1145–1160. 10.1007/s40520-023-02409-8 (2023). 37160649 10.1007/s40520-023-02409-8 PMC10169152

[78] Hernandez-Ruiz, V. et al. Infectious diseases and cognition: Do we have to worry?. *Neurol. Sci.* **43**, 6215–6224. 10.1007/s10072-022-06280-9 (2022). 35867217 10.1007/s10072-022-06280-9 PMC9305033

[79] Panza, F. et al. Time to test antibacterial therapy in Alzheimer’s disease. *Brain* **142**, 2905–2929. 10.1093/brain/awz244 (2019). 31532495 10.1093/brain/awz244

[80] Carneiro, V. C. S., Pereira, J. G. & de Paula, V. S. Family Herpesviridae and neuroinfections: Current status and research in progress. *Mem. Inst. Oswaldo Cruz* **117**, e220200. 10.1590/0074-02760220200 (2022). 36417627 10.1590/0074-02760220200 PMC9677594

[81] Licastro, F. & Porcellini, E. Activation of endogenous retrovirus, brain infections and environmental insults in neurodegeneration and Alzheimer’s disease. *Int. J. Mol. Sci.* **22**, 7263. 10.3390/ijms22147263 (2021). 34298881 10.3390/ijms22147263 PMC8303979

[82] James, L. M. & Georgopoulos, A. P. Immunogenetics of longevity and its association with human endogenous retrovirus K. *Front. Aging* **6**, 1471202. 10.3389/fragi.2025.1471202 (2025). 39967996 10.3389/fragi.2025.1471202 PMC11832543

[83] Mahley, R. W. & Huang, Y. Apolipoprotein E sets the stage: Response to injury triggers neuropathology. *Neuron* **76**, 871–885. 10.1016/j.neuron.2012.11.020 (2012). 23217737 10.1016/j.neuron.2012.11.020 PMC4891195

[84] Lopez-Lee, C., Torres, E. R. S., Carling, G. & Gan, L. Mechanisms of sex differences in Alzheimer’s disease. *Neuron* **112**(8), 1208–1221. 10.1016/j.neuron.2024.01.024 (2024). 38402606 10.1016/j.neuron.2024.01.024 PMC11076015

[85] Klein, S. L. & Flanagan, K. L. Sex differences in immune responses. *Nat. Rev. Immunol.* **16**(10), 626–638 (2016). 27546235 10.1038/nri.2016.90

[86] Nasreddine, Z. S. et al. The Montreal Cognitive Assessment, MoCA: A brief screening tool for mild cognitive impairment. *J. Am. Geriatr. Soc.* **53**, 695–699. 10.1111/j.1532-5415.2005.53221.x (2005). 15817019 10.1111/j.1532-5415.2005.53221.x

[87] Reymer, P. W., Groenemeyer, B. E., Van de Burg, R. & Kastelein, J. J. Apolipoprotein E genotyping on agarose gels. *Clin. Chem.* **41**, 1046–1047 (1995). 7600689

[88] Cano, P. et al. Common and well-documented HLA alleles: Report of the Ad-Hoc committee of the American Society for Histocompatiblity and Immunogenetics. *Hum. Immunol.* **68**, 392–417. 10.1016/j.humimm.2007.01.014 (2007). 17462507 10.1016/j.humimm.2007.01.014

[89] Reynisson, B., Alvarez, B., Paul, S., Peters, B. & Nielsen, M. NetMHCpan-4.1 and NetMHCIIpan-4.0: Improved predictions of MHC antigen presentation by concurrent motif deconvolution and integration of MS MHC eluted ligand data. *Nucleic Acids Res.* **48**, W449–W454. 10.1093/nar/gkaa379 (2020). 32406916 10.1093/nar/gkaa379 PMC7319546

[90] Charonis, S., Tsilibary, E. P. & Georgopoulos, A. SARS-CoV-2 virus and human leukocyte antigen (HLA) Class II: Investigation in silico of binding affinities for COVID-19 protection and vaccine development. *J. Immunol. Sci.* **4**, 12–23. 10.29245/2578-3009/2020/4.1198 (2020). 40370340 10.29245/2578-3009/2020/4.1198 PMC12077080

[91] Charonis, S. A., Tsilibary, E. P. & Georgopoulos, A. P. In silico investigation of binding affinities between human leukocyte antigen class I molecules and SARS-CoV-2 virus spike and ORF1ab proteins. *Explor. Immunol.* **1**, 16–26. 10.37349/ei.2021.00003 (2021). 40746829 10.37349/ei.2021.00003 PMC12311910

[92] Chicz, R. M. et al. Predominant naturally processed peptides bound to HLA-DR1 are derived from MHC-related molecules and are heterogeneous in size. *Nature* **358**, 764–768. 10.1038/358764a0 (1992). 1380674 10.1038/358764a0

[93] Engelhard, V. H. Structure of peptides associated with class I and class II MHC molecules. *Annu. Rev. Immunol.* **12**, 181–207. 10.1146/annurev.iy.12.040194.001145 (1994). 7516668 10.1146/annurev.iy.12.040194.001145

[94] Omran, A., Amberg, A. & Ecker, G. F. Exploring diverse approaches for predicting interferon-gamma release: Utilizing MHC class II and peptide sequences. *Brief. Bioinform.*10.1093/bib/bbaf101 (2025). 40067115 10.1093/bib/bbaf101 PMC11894801

[95] Istrail, S. et al. Comparative immunopeptidomics of humans and their pathogens. *Proc. Natl. Acad. Sci. U.S.A.* **101**, 13268–13272. 10.1073/pnas.0404740101 (2004). 15326311 10.1073/pnas.0404740101 PMC516558

[96] Mahan, M. Y., Leuthold, A. C. & Georgopoulos, A. P. Spatiotemporal brain network analysis of healthy humans based on magnetoencephalography and functional MRI in the resting state. *BMC Neurosci.* **16**(Suppl 1), 155. 10.1186/1471-2202-16-S1-P155 (2015).

[97] Box, G. E., Jenkins, G. M., Reinsel, G. C. & Ljung, G. M. *Time Series Analysis: Forecasting and Control* (Wiley, 2015).

[98] Fisher, R. A. *Statistical Methods for Research Workers* 13th edn. (Oliver & Boyd, 1958).

[99] Paternoster, R., Brame, R., Mazerolle, P. & Piquero, A. Using the correct statistical test for the equality of regression coefficients. *Criminology* **36**(4), 859–866. 10.1111/j.1745-9125.1998.tb01268.x (1998).

