# Supplementary material for: A positive association between phosphorylated Tau217 (pTau217) and neural correlations is prevented by human leukocyte antigen allele DRB1*13:01
Source: Sci Rep. 2026 Mar 26;16:15026. doi: 10.1038/s41598-026-44894-7 (PMC13171920; doi:10.1038/s41598-026-44894-7)
Supplement: Supplementary file 1 — Supplementary Material 1 [file 41598_2026_44894_MOESM1_ESM.docx]

Table S1. Amino acid sequences of HHV1 and HERVK viruses that affected the pTau217-SNI association. Strain labels are from Uniprot (https://www.uniprot.org/uniprotkb/).

| HHV1: Q69091 · GD_HHV11 | Envelope glycoprotein D | 394 AA |
| --- | --- | --- |

MGGAAARLGAVILFVVIVGLHGVRSKYALVDASLKMADPNRFRGKDLPVLDQLTDPPGVRRVYHIQAGLPDPFQPPSLPITVYYAVLERACRSVLLNAPSEAPQIVRGASEDVRKQPYNLTIAWFRMGGNCAIPITVMEYTECSYNKSLGACPIRTQPRWNYYDSFSAVSEDNLGFLMHAPAFETAGTYLRLVKINDWTEITQFILEHRAKGSCKYALPLRIPPSACLSPQAYQQGVTVDSIGMLPRFIPENQRTVAVYSLKIAGWHGPKAPYTSTLLPPELSETPNATQPELAPEDPEDSALLEDPVGTVAPQIPPNWHIPSIQDAATPYHPPATPNNMGLIAGAVGGSLLAALVICGIVYWMRRHTQKAPKRIRLPHIREDDQPSSHQPLFY

| Human endogenous retrovirus group K P10266 | 10 Pol protein | 1014 AA |
| --- | --- | --- |

NKSRKRRNRVSFLGAVTVEPPKPIPLTWKTEKPVWVNQWPLPKQKLEALHLLANEQLEKGHIEPSFSPWNSPVFVIQKKSGKWHTLTDLRAVNAVIQPMGPLQPGLPSPAMIPKDWPLIIIDLKDCFFTIPLAEQDCEKFAFTIPAINNKEPATRFQWKVLPQGMLNSPTICQTFVGRALQPVREKFSDCYIIHYIDDILCAAETKDKLIDCYTFLQAEVANAGLAIASDKIQTSTPFHYLGMQIENRKIKPQKIEIRKDTLKTLNDFQKLLGDINWIRPTLGIPTYAMSNLFSILRGDSDLNSQRILTPEATKEIKLVEEKIQSAQINRIDPLAPLQLLIFATAHSPTGIIIQNTDLVEWSFLPHSTVKTFTLYLDQIATLIGQTRLRITKLCGNDPDKIVVPLTKEQVRQAFINSGAWQIGLANFVGLIDNHYPKTKIFQFLKLTTWILPKITRREPLENALTVFTDGSSNGKAAYTGPKERVIKTPYQSAQRDELVAVITVLQDFDQPINIISDSAYVVQATRDVETALIKYSMDDQLNQLFNLLQQTVRKRNFPFYITYIRAHTNLPGPLTKANEQADLLVSSALIKAQELHALTHVNAAGLKNKFDVTWKQAKDIVQHCTQCQVLHLPTQEAGVNPRGLCPNALWQMDVTHVPSFGRLSYVHVTVDTYSHFIWATCQTGESTSHVKKHLLSCFAVMGVPEKIKTDNGPGYCSKAFQKFLSQWKISHTTGIPYNSQGQAIVERTNRTLKTQLVKQKEGGDSKECTTPQMQLNLALYTLNFLNIYRNQTTTSAEQHLTGKKNSPHEGKLIWWKDNKNKTWEIGKVITWGRGFACVSPGENQLPVWLPTRHLKFYNEPIGDAKKRASTEMVTPVTWMDNPIEVYVNDSIWVPGPIDDRCPAKPEEEGMMINISIGYRYPPICLGRAPGCLMPAVQNWLVEVPTVSPISRFTYHMVSGMSLRPRVNYLQDFSYQRSLKFRPKGKPCPKEIPKESKNTEVLVWEECVANSAVIL
